# Esculentoside A mitigates oxidative stress and neuronal apoptosis in spinal cord injury by modulating the Nrf2/HO-1 pathway

**DOI:** 10.3389/fneur.2026.1861879

**Published:** 2026-06-24

**Authors:** Guoqing Zhu, Weiwei Li, Mengge Sun, Jingyu Yan, Wei Hu

**Affiliations:** Laboratory Department, Shanghai Municipal Hospital of Traditional Chinese Medicine, Shanghai University of Traditional Chinese Medicine, Shanghai, China

**Keywords:** Esculentoside A, neuronal apoptosis, Nrf2/HO-1 pathway, oxidative stress, spinal cord injury

## Abstract

**Objective:**

Spinal cord injury (SCI) is a profoundly disabling condition affecting the central nervous system. Neuronal apoptosis constitutes a critical pathological event leading to neurological dysfunctions, which is further exacerbated by oxidative stress following SCI. Esculentoside A (EsA), a bioactive saponin isolated from Phytolaca esculenta, exhibits neuroprotective potential in our preliminary studies. However, whether EsA attenuates oxidative stress and neuronal apoptosis in SCI remains unclear.

**Purpose:**

The current study aimed to investigate the protective potential of EsA against oxidative stress and neuronal apoptosis following SCI, and to elucidate the associated molecular mechanisms.

**Methods:**

SCI was modeled in rats via contusion using the PSI-IH 0400 Striker impactor, and rats were treated intraperitoneally with 10 mg/kg EsA once daily. The Basso, Beattie, and Bresnahan (BBB) scale, grid walk analysis, and footprint test were adopted to evaluate motor function dynamically. Histopathological alterations in spinal cord tissue were examined by Hematoxylin-eosin (HE), Luxol Fast Blue (LFB), and Nissl staining. Oxidative stress markers, including hydrogen peroxide (H_2_O_2_) and malondialdehyde (MDA), along with antioxidant enzymes glutathione peroxidase (GSH-PX) and superoxide dismutase (SOD), were quantified in spinal cord homogenates using commercial assay kits. Western blot, immunofluorescence staining, and molecular docking were employed to investigate the underlying mechanisms.

**Results:**

EsA significantly improved motor function and reduced histopathological damage in SCI rats. This neuroprotective effect was accompanied by a significant improvement in oxidative stress biomarkers and neuronal apoptosis in the injured spinal cord, coinciding with activation of the nuclear factor erythroid 2-related factor 2 (Nrf2)/heme oxygenase-1 (HO-1) pathway.

**Conclusions:**

In conclusion, EsA exerts a neuroprotective effect against SCI by modulating oxidative stress and neuronal apoptosis partially through activation of the Nrf2/HO-1 pathway, indicating its promise as a therapeutic agent for SCI.

## 1 Introduction

Spinal cord injury (SCI) is a debilitating neurological trauma that leads to significant long-term disability and increased mortality, posing a major global public health challenge (1). Despite advancements in clinical management, conventional pharmacotherapies for SCI have limited efficacy and are frequently associated with considerable adverse effects (2, 3). Therefore, deciphering the pathophysiological mechanisms underlying SCI and developing more effective therapeutic strategies remain critical unmet needs in neurotrauma research.

SCI evolves through an irreversible primary lesion resulting from direct physical trauma, followed by a prolonged secondary injury phase (4). This latter stage is propagated by a cascade of interconnected pathological events, including vasogenic edema, oxidative stress, neuroinflammation, and neuronal apoptosis, collectively representing a critical window for neuroprotective interventions (5). Mounting evidence has implicated oxidative stress and neuronal apoptosis as pivotal mediators of spinal cord dysfunction post-SCI (6, 7). Oxidative stress is mainly triggered by an overproduction of reactive oxygen species (ROS), leading to damage of vital biomolecules (proteins, lipids, and DNA) (8). After SCI, activated microglia generate substantial amounts of ROS, impairing neuronal integrity and further exacerbating neuronal apoptosis (9). The loss of neurons through apoptosis impairs neural circuitry, resulting in motor, sensory, and autonomic functional deficits (10). Consequently, therapeutic strategies targeting oxidative stress and neuronal apoptosis represent promising avenues for improving neurological outcomes after SCI.

Nuclear factor erythroid 2-related factor 2 (Nrf2) functions as a master modulator of the intracellular antioxidant defensive machinery, playing a crucial role in sustaining redox balance (11). Oxidative stress triggers Nrf2 to upregulate the expression of diverse cytoprotective enzymes, with heme oxygenase-1 (HO-1) and NAD(P)H: quinone oxidoreductase 1 (NQO1) as the primary targets (12). These enzymes efficiently scavenge excess ROS and safeguard cellular components against oxidative damage (13). Notably, both genetic and pharmacological activation of Nrf2 have been demonstrated to alleviate oxidative damage and neuronal loss in multiple preclinical models of SCI. For example, Nrf2-knockout mice have displayed exacerbated oxidative stress, increased neuronal apoptosis, and worse functional recovery after spinal cord trauma compared with wild-type controls (14). In contrast to genetic depletion, treatment with the Nrf2 activator dimethyl fumarate (DMF) has significantly reduced MDA accumulation, enhanced Bcl-2 expression, and preserved neuronal survival in a rat model of traumatic SCI (15). Pharmacologically, natural compounds such as curculigoside have also been reported to alleviate oxidative injury and neuronal apoptosis in SCI by targeting the Nrf2/HO-1 pathway (16). Collectively, these findings imply that enhancing the Nrf2/HO-1 axis exerts neuroprotection in SCI by suppressing oxidative damage and apoptotic neuronal loss.

Natural monomers derived from traditional Chinese medicinal herbs have emerged as attractive neuroprotective candidates for managing diverse central nervous system (CNS) pathologies, owing to their minimal toxicity and a favorable safety profile (17). Esculentoside A (EsA), a natural saponin isolated from the roots of *Phytolaca esculenta*, exhibits potent antioxidant, anti-apoptotic, and anti-inflammatory activities (18, 19). Previous studies have shown that EsA activates the Nrf2 pathway to mitigate oxidative stress (20). Furthermore, EsA has improved cognitive function by suppressing neuronal apoptosis in murine models of Alzheimer's disease (21). Notably, our preliminary findings have demonstrated that EsA exerts neuroprotective actions in SCI rats (22). However, the specific molecular mechanism by which EsA alleviates SCI remains unclear, particularly whether it targets oxidative damage and neuronal apoptosis by directly modulating the Nrf2/HO-1 pathway.

This study utilized a rat contusion model of SCI and administered EsA through intraperitoneal injection to assess its therapeutic potential. EsA treatment significantly reduced tissue cavitation, enhanced myelination, and improved motor function in rats with SCI. Mechanistically, the neuroprotection conferred by EsA was associated with enhanced activity of the Nrf2/HO-1 pathway, which in turn alleviated oxidative stress and neuronal apoptosis.

## 2 Methods

### 2.1 Rats

Sprague-Dawley rats, female and aged 6–8 weeks (200–220 g), were maintained in a suitable environment for 3–5 days of acclimatization before experiments. All animal procedures were sanctioned by the Institutional Ethics Committee of Municipal Traditional Chinese Medicine Hospital Affiliated to Shanghai University of Traditional Chinese Medicine. We implemented all essential steps to reduce animal usage and alleviate their distress.

### 2.2 Pharmacological treatments and experimental groups

**Figure 1:**
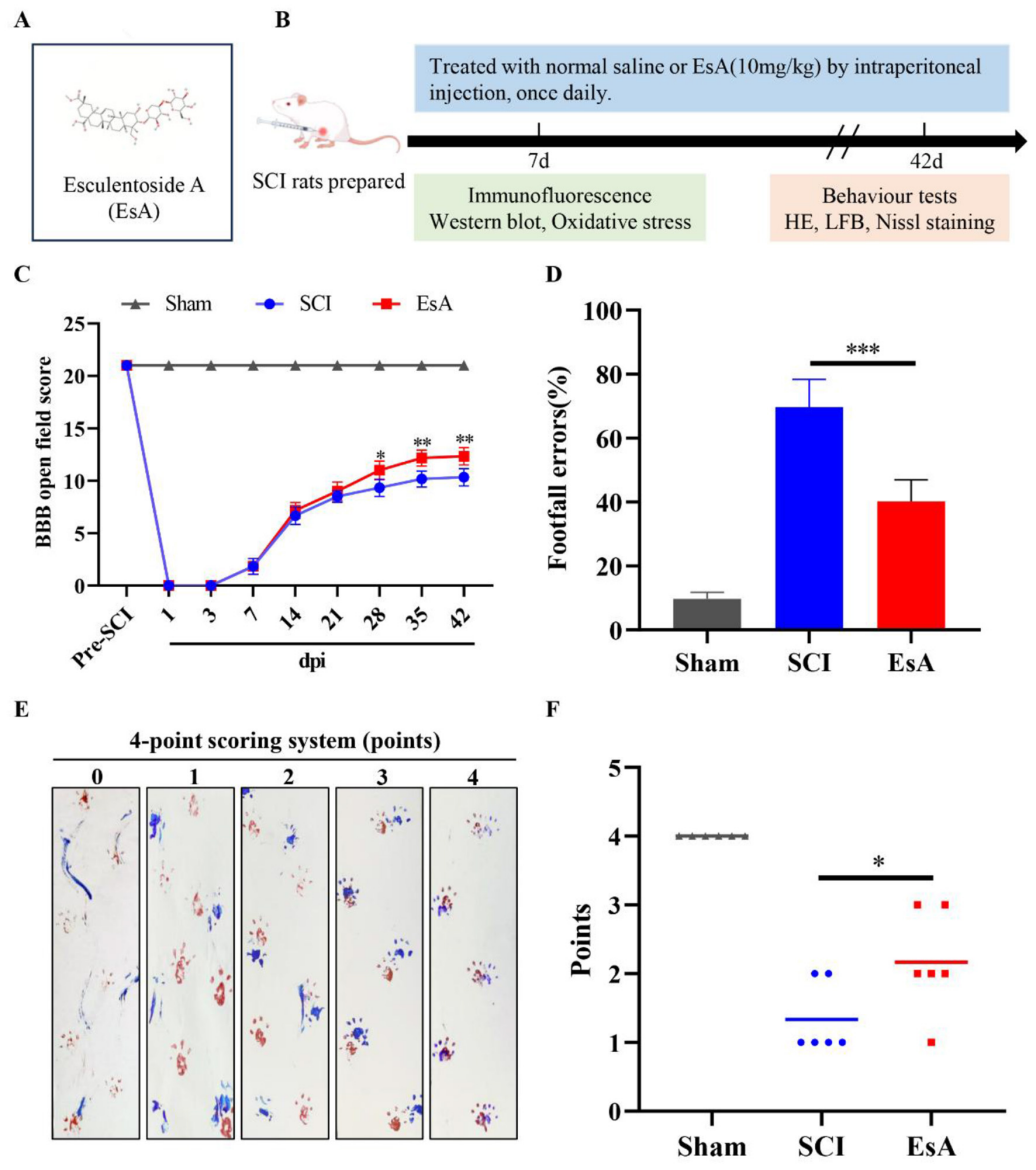
EsA ameliorated locomotor function recovery in SCI rats. **(A)** Chemical information of EsA. **(B)** Pattern program of the animal experimentation design. **(C)** BBB scores: The BBB scores were assessed from postoperative day 1 through week 6 following SCI. **(D)** The grid walk assay was conducted at the 6-week post-injury timepoint. **(E, F)** footprint test at 6 weeks after injury. Values are presented as mean ± SD (*n* = 6 per group). *p < 0.05, **p < 0.01, ***p < 0.001 vs. SCI group (Anova).

Esculentoside A (Figure 1A, purity >99.8%, Shanghai Yuanye Biotechnology, China) was dissolved in sterile physiological saline. All rats were randomly allocated into three groups: Sham (Sham, operation without SCI), SCI (SCI, intraperitoneal injection of sterile saline), and EsA (SCI, intraperitoneal injection of EsA at 10 mg/kg). Animal groups were randomly assigned using computer-generated numbers, and allocation concealment was ensured with sealed opaque envelopes. All experiments were performed with 6 biological replicates (*n* = 6 rats per group). Rats received daily intraperitoneal injections of EsA or saline throughout the experimental period. The dose of 10 mg/kg was determined according to our preliminary study and previous reports demonstrating neuroprotective efficacy without toxicity in rodent models (22). The experimental design is shown in Figure 1B. All outcome assessments, including behavioral tests, histological quantification, Western blot analysis, and immunofluorescence imaging, were performed by investigators blinded to the experimental groups. For Western blot, immunofluorescence analyses, and oxidative stress biomarker detection, 3 technical replicates were conducted per biological sample to ensure reproducibility and reliability. All data were analyzed by a third researcher who was also unaware of the treatment groups to minimize observer bias.

### 2.3 SCI model procedures

SCI was induced in rats using a well-established contusion protocol from our previous study (23). In this study, the PSI-IH 0400 Striker (PSI, Las Vegas, NV, USA) was employed to establish a T9 segment contusion injury model in rats. Rats in the experiments were anesthetized using intraperitoneal pentobarbital (50 mg/kg). We carried out a laminectomy at the T9 segment to observe the dorsal spinal cord. A moderate spinal cord contusion was induced using the PSI-IH striker, and the occurrence of tail flicking and hindlimb spasms was considered as the model was successfully established. The surgical area was disinfected with povidone-iodine, followed by suturing the skin and muscles on the rat's back. Postoperative care included manual bladder expression three times daily until spontaneous micturition recovered, and subcutaneous injection of antibiotics once daily for 7 days to prevent infection. Animals that failed to meet the modeling standard, suffered from severe postoperative infection, unexpected death, or obvious physical abnormality during the experiment were excluded from subsequent analyses.

### 2.4 Motor function tests

After undergoing the above treatment, motor recovery in SCI rats was evaluated using the BBB functional score, grid walk analysis, and footprint test.

The BBB locomotor scale was used to assess the motor recovery of their hind limbs. The SCI rats underwent a 5-min habituation period in the open field. Two researchers scored each rat's hindlimb motor function using a rating scale at specific time points, with the average of their scores serving as the final score.

The grid walk test was used to assess hindpaw placement control in rats with SCI. The experimental setup for the test consisted of a 1-square-meter metal mesh plate that was divided into meshes of varying sizes. Two researchers simultaneously recorded the total number of hindlimb steps and the frequency of falls from the grid when the rats walked on it, and then calculated the hindpaw placement error rate.

The footprint test involved applying red ink to the forepaws and blue ink to the hindpaws of rats, respectively. Rats were allowed to traverse a dark corridor with white paper-lined floors to record their footprints, which were subsequently analyzed for gait parameters. The scoring system was used as previously described (24).

### 2.5 Tissue preparation

SCI rats were anesthetized and intracardially perfused sequentially with phosphate-buffered saline (PBS, pH 7.4) and subsequently with 4% paraformaldehyde (PFA) at 7 and 42 days after SCI, respectively. Spinal cord segments flanking the injury site (5 mm rostral and 5 mm caudal) were harvested and immersed in 30% sucrose solution or 4% PFA solution for dehydration. Spinal cord tissues were cryopreserved and mounted in optimal cutting temperature (OCT, TissueTek, Miles, Elkart, IN) compound for cryosectioning. Spinal tissue slices were prepared for histological analysis and immunofluorescence staining.

### 2.6 Histological analysis

Frozen sections from 6-week post-SCI tissues were subjected to hematoxylin-eosin (HE) (Solarbio, China), LFB (Sigma, St. Louis, Missouri, USA), and Nissl (Solarbio, China) staining. Lesion area and myelin integrity within the epicenter and 1–4 mm rostral/caudal to the injury site were evaluated by HE and LFB staining. Damage and myelin-sparing areas were quantified using ImageJ, and data were normalized to the entire stained area and presented as percentages. Nissl staining was performed to visualize ventral motor neurons (VMNs) in the spinal cord, with surviving neurons quantified by counting Nissl-positive profiles. Staining methods and evaluation were performed as previously reported (25).

### 2.7 Immunofluorescence

Frozen spinal cord sections from 7-day post-SCI rats were air-dried, blocked with 10% goat serum containing 0.1% Triton X-100 (Sigma) for 60 min, and probed with primary antibodies overnight. Following PBS washes (3 × 5 min), sections were incubated with secondary antibodies for 60 min under dark conditions, followed by DAPI (Sigma-Aldrich, St. Louis, Missouri, USA; 2 μg/mL) counterstaining. Five intact tissue slices from each rat were evaluated blindly. Positive cells were counted in five consecutive sections spanning the rostrodorsolateral to caudoventromedial regions that covered the injury epicenter. Primary antibodies against NeuN (Ab104224, at 1:300, Abcam, Cambridge, United Kingdom), cysteine-dependent aspartate-specific protease-3 (Caspase-3, Ab13847, at 1:300, Abcam, Cambridge, United Kingdom), CD68 (ab125212, at 1:500 Abcam, Cambridge, United Kingdom), and Nrf2 (80593-1-RR, at 1:500, Proteintech, Wuhan, Hubei, China) were used.

### 2.8 Western blot analysis

Spinal cord perilesional samples (5 mm rostral/caudal to the lesion epicenter) obtained from 7-day post-SCI rats were homogenized in ice-cold RIPA lysis buffer (Beyotime) containing a protease inhibitor (1:50, Beyotime). Protein concentrations were measured by employing a bicinchoninic acid (BCA) assay kit (Beyotime). SDS-PAGE resolved identical amounts of protein from each sample and subsequently electrotransferred them onto PVDF membranes. After blocking, membranes were incubated overnight at 4 °C with primary antibodies, then washed and incubated with secondary antibodies. Immunoreactive bands were visualized using ECL reagent (Thermo Fisher Scientific, Waltham, Massachusetts, USA) and recorded digitally via the ChemiDoc system (Bio-Rad, Hercules, California, USA). The gray values were obtained from ImageJ software (Bio-Rad, Hercules, California, USA). The expression levels of target proteins are presented as the ratio of target protein intensity to β-actin intensity, and all target protein levels were normalized to β-actin expression to account for loading variations. The following primary antibodies were employed: B cell lymphoma 2 (Bcl-2, ab194583, at 1:1000), Bcl-2-associated X protein (Bax, ab194583, at 1:1000), Cleaved Caspase-3 (C-Caspase-3, ab32042, at 1:1000), Nrf2 (Ab62352, at 1:1000), HO-1(Ab189491, at 1:1000), NAD(P)H: quinone oxidoreductase 1 (NQO-1, Ab221215, at 1:1000), and β-actin (Ab8226, at 1:1000, all from Abcam). All Western blot results were obtained from full-length blots. No cropped regions were selectively imaged, and full-length blot images are available upon request from the authors.

### 2.9 Analysis of oxidation products

Spinal cord tissue segments (1 cm in length) were collected from both the rostral and caudal sides of the injury epicenter at predetermined time points post-injury. Following homogenization in saline, samples underwent centrifugation at 1,000 × g for 10 min at 4 °C, and the supernatants were obtained for subsequent assays. The levels of hydrogen peroxide (H_2_O_2_) and malondialdehyde (MDA) were assessed with commercially available kits (Nanjing Jiancheng, China).

### 2.10 Determination of antioxidant enzyme activity

The activities of glutathione peroxidase (GSH-PX) and superoxide dismutase (SOD) were quantified in the collected supernatants at selected time points post-injury. The assays were performed using commercial kits specific for SOD and GSH-PX (both from Nanjing Jiancheng, China).

### 2.11 Molecular docking

The EsA structure was obtained from PubChem and subsequently converted into a 3D conformation using Chem3D software. The 3D complex structures of Nrf2/Kelch-like ECH-associated protein 1 (Keap1) were downloaded from the PDB database (PDB ID: 1X2R) (26, 27). Molecular preprocessing of EsA and Nrf2/Keap1 was conducted using AutoDock Vina (28). Docking parameters were subsequently defined to ensure the docking box fully encompassed both the ligand and the receptor. The binding affinity between EsA and the Nrf2/Keap1 complex was calculated using AutoDock Vina, and the highest predicted affinity was subsequently visualized and analyzed with PyMOL.

### 2.12 Statistical analysis

SPSS 26.0 was adopted to conduct data analysis. Data normality was assessed using the Shapiro-Wilk test before parametric analyses. Two-way analysis of variance (ANOVA) with Tukey's post hoc test for pairwise comparison was applied to evaluate the data involving both time and group variables. One-way ANOVA or the Kruskal-Wallis non-parametric test (as appropriate) was applied for the analysis of other data, with Bonferroni's *post-hoc* test for multiple comparisons. All multiple comparison corrections were applied consistently across all analyses. Statistical differences were defined as significant at *p* < 0.05.

## 3 Results

### 3.1 EsA improved motor function recovery after SCI

Spinal cord contusion was induced in rats, and the animals were treated with EsA or saline as described in Methods. Behavioral assessments of SCI rats were conducted using the BBB scale, grid walk analysis, and footprint test. During the first three weeks of administration, the BBB scores at different time points exhibited no substantial differences between the EsA and SCI groups. Notably, the EsA group consistently showed significantly higher BBB scores relative to the SCI group between weeks 4 and 6 post-injury (Figure 1C). Grid walk analysis revealed a lower hindpaw placement error rate in the EsA group compared to the SCI group at 6 weeks post-injury (Figure 1D). Concomitantly, the SCI group exhibited lower footprint test scores relative to the sham group, while EsA treatment significantly reversed this gait impairment (Figures 1E, F). Collectively, these results indicated that EsA improved locomotor performance in rats following SCI.

### 3.2 EsA alleviated histological damage

**Figure 2:**
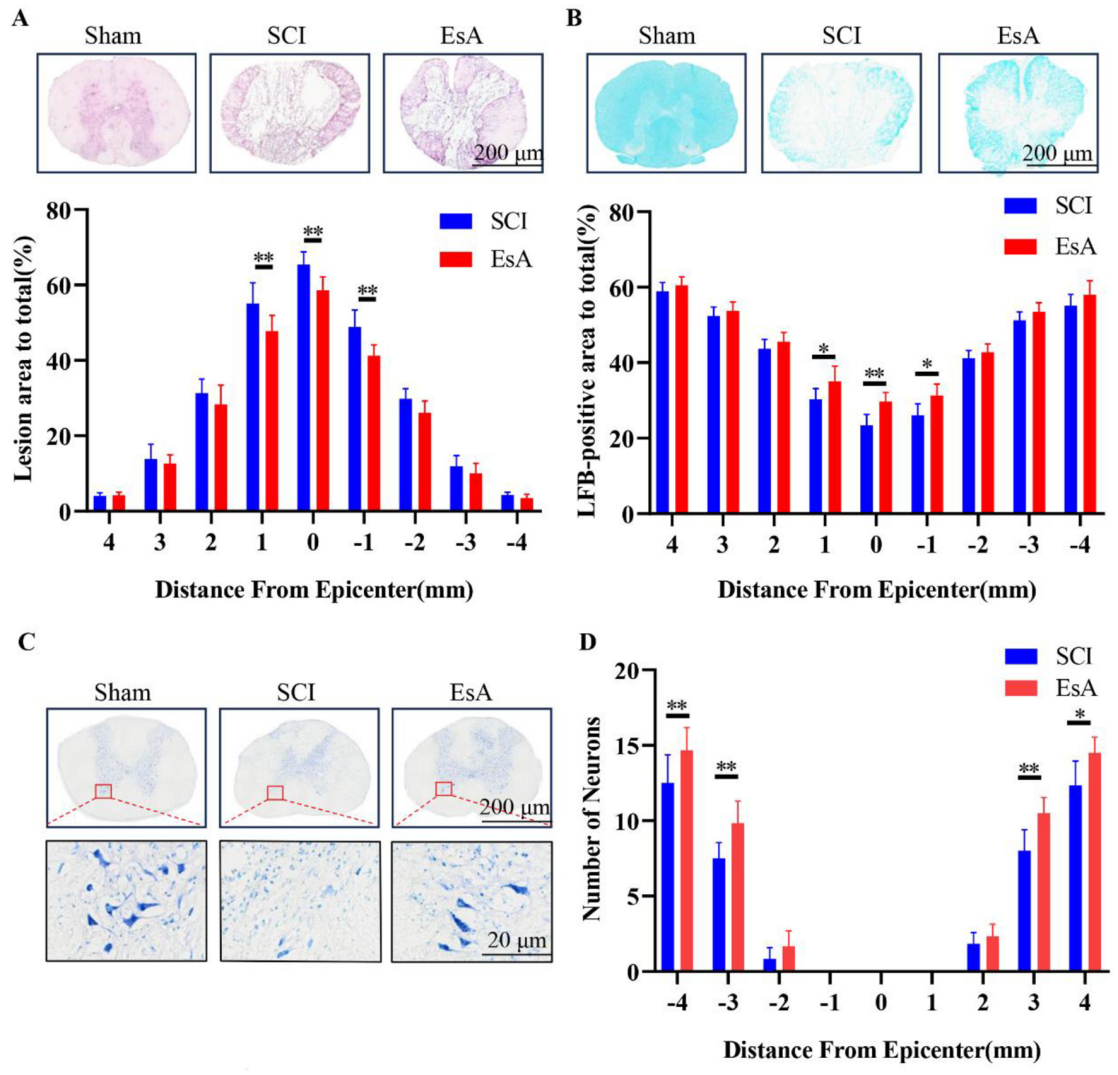
EsA alleviated histopathological damage and promoted tissue repair following SCI. **(A)** Typical pictures of HE staining and the lesion area of the SCI and EsA groups. **(B)** Typical LFB-stained images illustrating residual myelination differences between the SCI and EsA groups. **(C, D)** Comparison of VMNs in the SCI and EsA groups using representative Nissl staining images. Values are presented as mean ± SD (*n* = 6 per group). *p < 0.05, **p < 0.01 vs. SCI group (Anova).

To evaluate the impact of EsA on spinal cord histopathology after SCI, lesion size, myelinated white matter integrity, and surviving ventral motor neurons (VMNs) were assessed using HE, LFB, and Nissl staining, respectively. HE and LFB staining revealed that the EsA group had a smaller lesion area and increased myelin density compared to the SCI group at the epicenter and 1 mm rostral/caudal regions (Figures 2A, B). Nissl staining indicated a nearly complete loss of VMNs within the lesion core across all injured groups, and the survival rate of VMNs increased progressively with increasing distance from the lesion epicenter. At ±3 mm and ±4 mm from the lesion epicenter, the SCI group exhibited fewer surviving VMNs, whereas EsA treatment significantly attenuated this neuronal loss (Figures 2C, D).

These data confirmed that EsA contributed to histological preservation in SCI rats.

### 3.3 EsA attenuated neuronal apoptosis following SCI

**Figure 3:**
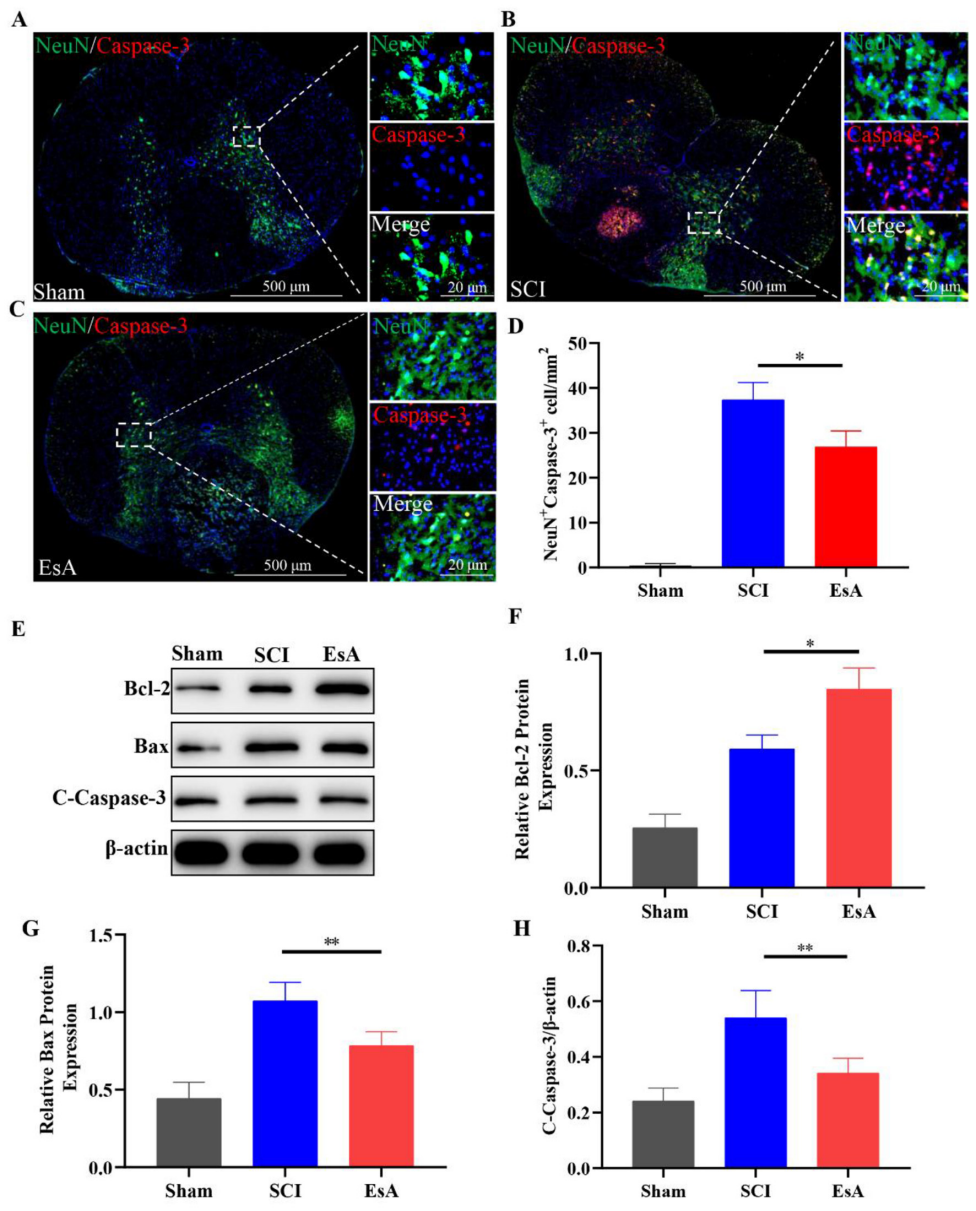
EsA inhibited apoptosis in the injured spinal cord. **(A–C)** NeuN/Caspase-3 double immunofluorescence in spinal cord sections. **(D)** Quantification of NeuN^+^/Caspase-3^+^ double-positive cells. **(E)** Protein expression of Bcl-2, Bax, and C-Caspase-3 by Western blot. **(F–H)** Quantitative results of Bcl-2, Bax, and C-Caspase-3 protein levels. Values are presented as mean ± SD (*n* = 6 per group). *p < 0.05, **p < 0.01 vs. SCI group (Anova).

To investigate the neuroprotective potential of EsA against neuronal apoptosis, immunofluorescence staining and Western blotting were employed. One week after SCI, double immunofluorescence staining for NeuN and Caspase-3 showed an increase in NeuN^+^/Caspase-3^+^ apoptotic neurons in the SCI group compared to the sham group. EsA treatment significantly decreased the number of double-positive cells (Figures 3A–D). Western blot analysis indicated that SCI activated apoptosis-associated proteins, with a marked upregulation of Bax and C-Caspase-3 and a significant downregulation of Bcl-2. EsA administration notably mitigated these changes, with significantly decreased Bax and C-Caspase-3 protein levels and elevated Bcl-2 expression compared with those in the SCI group (Figures 3E–H).

These results demonstrated that EsA inhibited apoptosis within the injured spinal cord tissue of SCI rats.

### 3.4 EsA ameliorated oxidative stress parameters in the spinal cord of SCI rats

**Figure 4:**
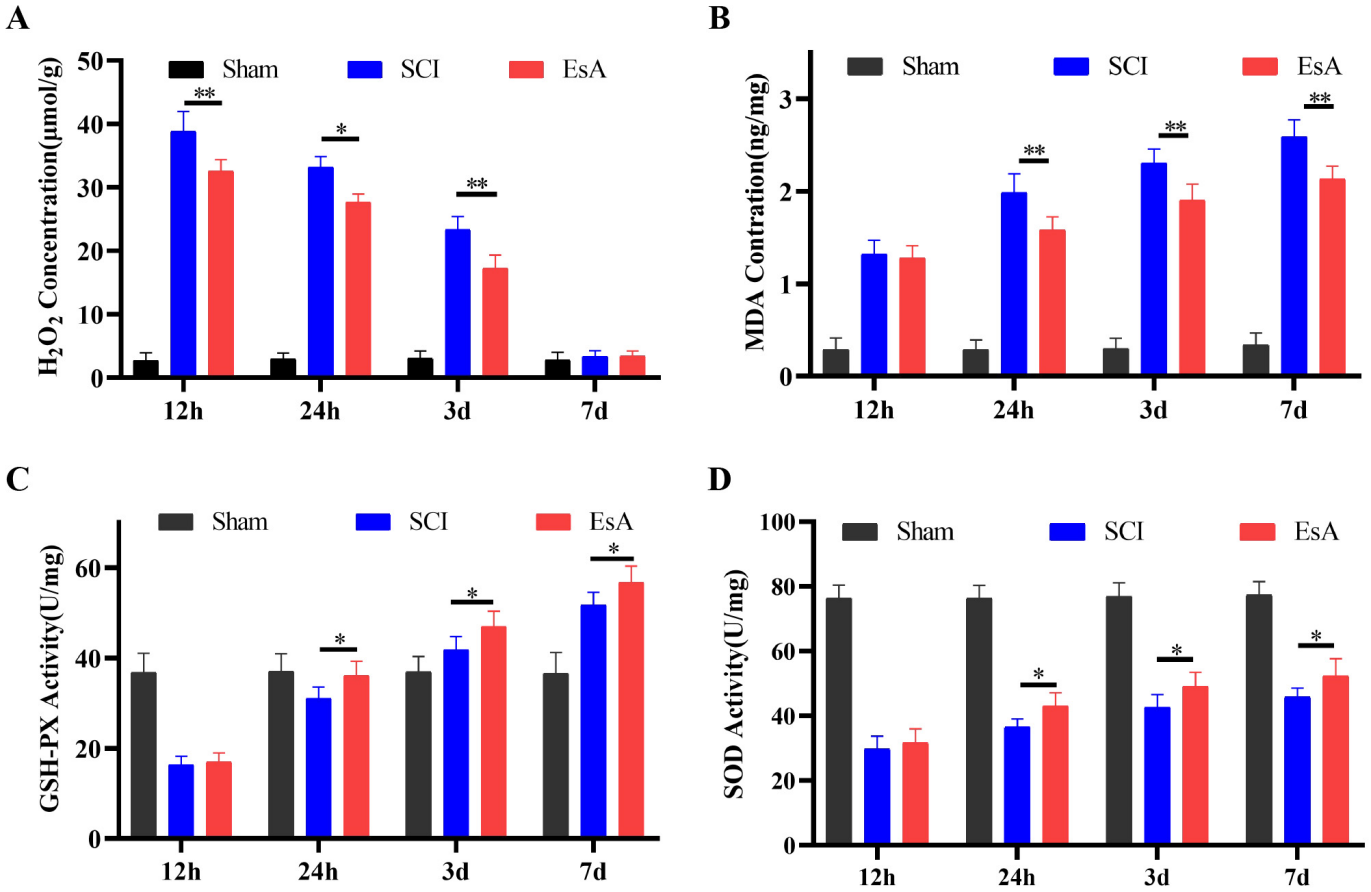
EsA modulated oxidative stress in the injured spinal cord. **(A, B)** Quantification of H_2_O_2_ and MDA levels. **(C, D)** Measurement of SOD and GSH-PX enzymatic activities. Values are presented as mean ± SD (*n* = 6 per group). *p < 0.05, **p < 0.01 vs. SCI group (Anova).

The impact of EsA on SCI-induced spinal cord oxidative stress was assessed by quantifying H_2_O_2_ and MDA levels, as well as GSH-PX and SOD activities. As shown in Figures 4A, B, H_2_O_2_ concentration in the spinal cord increased rapidly after SCI and subsequently gradually declined. At 12 h, 24 h, and 3 days post-injury, the EsA group had lower H_2_O_2_ levels compared to the SCI group. MDA levels were progressively increased after SCI, peaking at 7 days, and EsA administration significantly attenuated this elevation at 24 h, 3 days, and 7 days. GSH-PX and SOD enzymatic activities reached their lowest levels at 12 h post-SCI, then gradually recovered over time. Compared with the SCI group, EsA-treated rats exhibited significantly higher SOD and GSH-PX activities at 24 h, 3 days, and 7 days (Figures 4C, D).

The above results indicated that EsA's effectiveness against SCI appears to be associated with the modulation of oxidative stress.

### 3.5 Molecular docking between EsA and Nrf2

**Figure 5:**
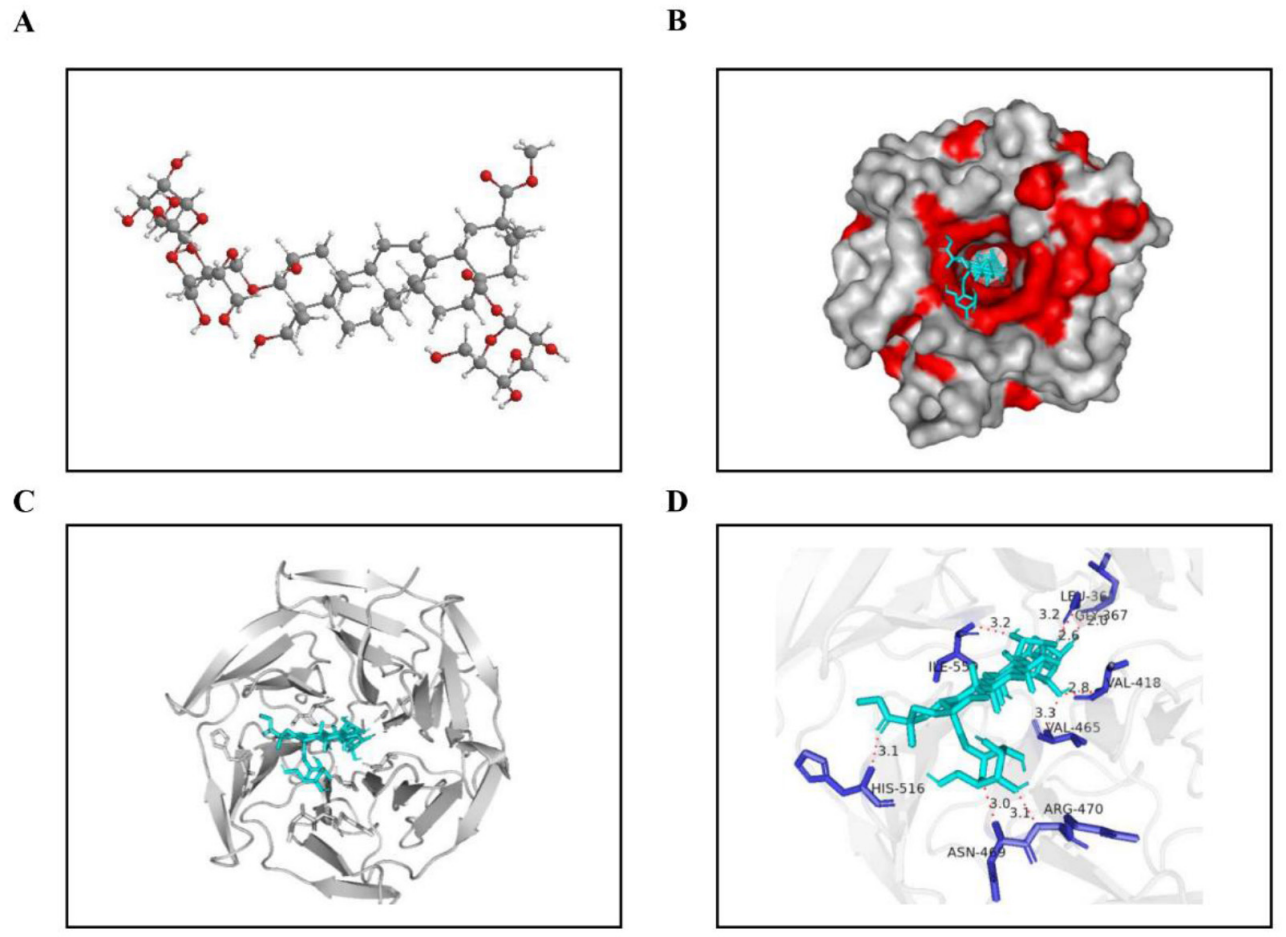
*In silico* molecular docking of EsA to the Nrf2/Keap1 complex. **(A)** 3D structures of EsA. **(B)** EsA was buried in the hydrophobic pocket of the Nrf2/Keap1 complex. **(C, D)** The binding models of EsA with the Nrf2/Keap1 complex.

To study the potential binding mode and affinity between EsA and Nrf2, molecular docking was conducted in AutoDock Vina according to standard protocols. The three-dimensional structure of EsA was illustrated in Figure 5A. The docking score between EsA and the Nrf2/Keap1 complex was −11.9 kcal/mol, indicating a favorable binding tendency of EsA toward the Nrf2/Keap1 complex *in silico*. As shown in Figure 5B, the red-highlighted residues are hydrophobic amino acids that fill the binding pocket at the EsA-Nrf2/Keap1 complex interface. Notably, the hydrogen bonds were established between EsA and key residues of the Nrf2/Keap1 complex, likely stabilizing the ligand-receptor interaction (Figures 5C, D). These *in silico* findings preliminarily suggested a potential molecular interaction between EsA and the Nrf2/Keap1 complex.

### 3.6 EsA modulated oxidative stress in rats with SCI accompanied by the activation of the Nrf2/HO-1 pathway

**Figure 6:**
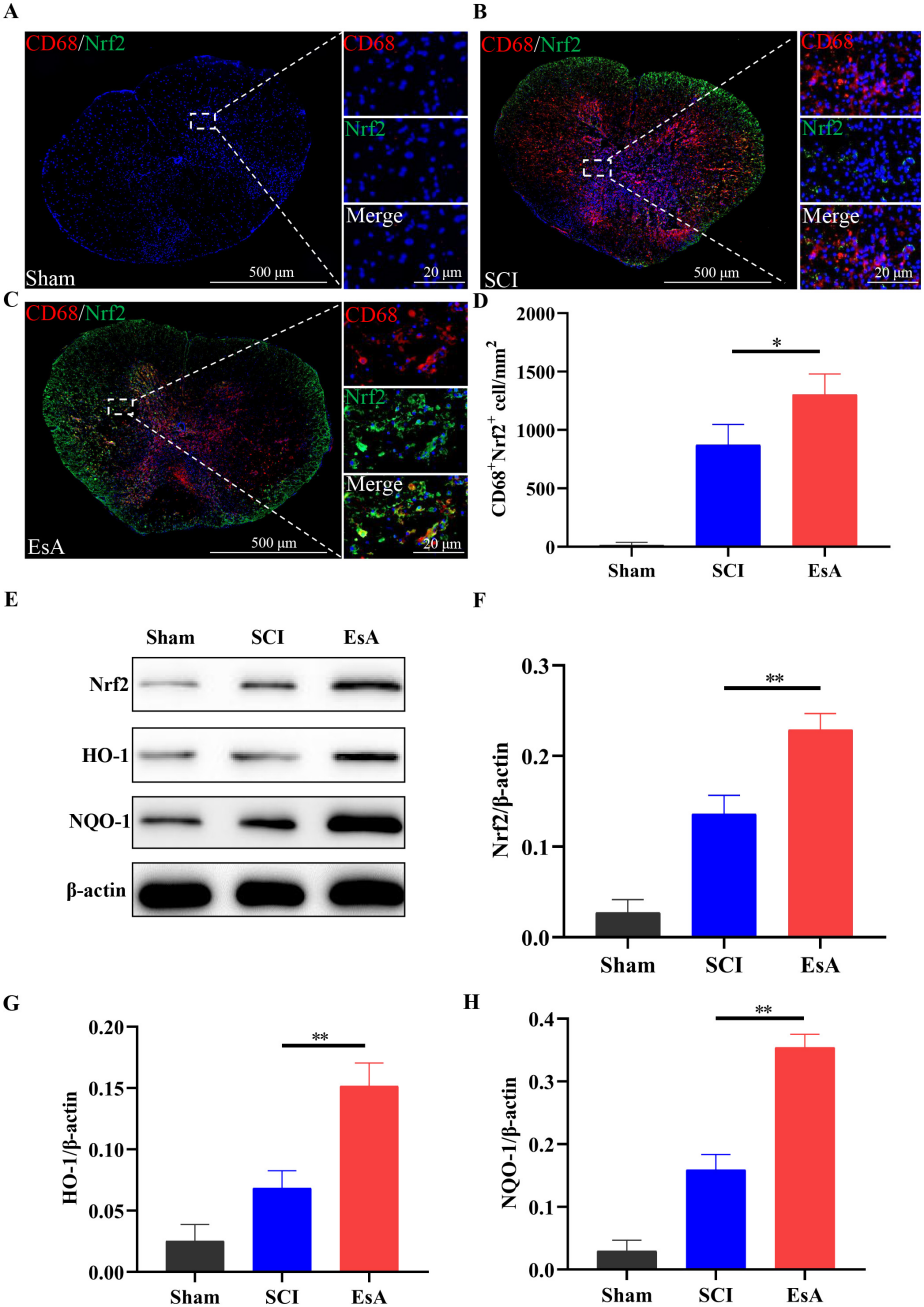
EsA mitigated oxidative stress following injury in association with Nrf2/HO-1 pathway activation. **(A–C)** Double-labeled images of CD68 (red) and Nrf2 (green) in injured spinal cord sections. **(D)** Quantification of CD68^+^/Nrf2^+^ double-positive cells. **(E–H)** Representative immunoblots and analysis of Nrf2, HO-1, and NQO-1 protein expression in injured spinal cord. Values are presented as mean ± SD (*n* = 6 per group). *p < 0.05, **p < 0.01 vs. SCI group (Anova).

Activated microglia rapidly generate substantial amounts of ROS post-SCI, which mediates oxidative stress in spinal cord tissues after SCI. Figures 6A–D illustrated that CD68^+^/Nrf2^+^ double-positive cells were absent in the sham group. In contrast, both the SCI and EsA groups showed an increase in these double-positive cells, and the EsA group exhibited a markedly higher abundance than the SCI group. In line with this cellular observation, protein levels of Nrf2, HO-1, and NQO-1 were upregulated in the SCI group vs. the sham group. Notably, EsA treatment further increased the expression of these antioxidant proteins, surpassing those measured in the SCI group (Figures 6E–H).

These findings confirmed that EsA regulated oxidative stress partially through triggering the Nrf2/HO-1 pathway.

## 4 Discussion

Despite being a major cause of permanent disability, effective pharmacological treatments for SCI remain inadequate (29). Consequently, there is an urgent imperative to develop novel and more efficacious therapeutics for SCI. The present study suggested that EsA facilitated locomotor functional recovery and ameliorated histopathological damage after SCI. Based on these findings, we further demonstrated that EsA mitigated oxidative stress and reduced neuronal apoptosis in the SCI model. Mechanistically, the neuroprotective effects of EsA are partially attributed to the increased activity of the Nrf2/HO-1 pathway.

SCI leads to impaired motor and sensory function caudal to the lesion, profoundly compromising patients' quality of life and independence in daily activities (30). In this study, we employed a rat model of traumatic SCI induced by the PSI-IH 0400 striker to evaluate the therapeutic potential of EsA. EsA treatment significantly improved the BBB score and reduced the hindpaw placement error rate in the grid walk analysis, while also reversing gait impairment in the footprint test compared to the SCI group, demonstrating its capacity to improve behavioral function. Notably, the restoration of locomotor function post-SCI is tightly linked to the extent of spinal cord tissue damage, the capacity for remyelination, and the preservation of VMNs, key structural determinants of neural circuitry integrity (31). Accordingly, we further evaluated the impact of EsA treatment on spinal cord histopathological changes post-injury. In accordance with the behavioral recovery data, HE and LFB staining demonstrated that EsA treatment mitigated tissue damage and maintained myelin integrity in the injured spinal cord. Additionally, EsA effectively protected VMNs from apoptotic loss. These structural and functional findings collectively validate the neuroprotective efficacy of EsA in SCI, providing a solid morphological and behavioral basis for its subsequent mechanistic exploration.

Neurons function as primary conduits for neural signal transmission, with axons providing the essential platform for myelination (32, 33). Given that neuronal loss severely hinders functional recovery after SCI, strategies aimed at neuronal survival hold significant therapeutic promise (34). Apoptosis, a type of regulated cell death dependent on caspases, is a key determinant of neurological outcome post-SCI (35, 36). Caspase-3, a central executioner protease of the caspase family, is markedly activated after SCI and plays a pivotal role in mediating neuronal apoptosis (37). Normally maintained as an inactive zymogen, caspase-3 undergoes proteolytic cleavage in response to apoptotic stimuli, thereby initiating the downstream execution phase of the apoptotic cascade. In the current study, SCI triggered a significant increase in Caspase3^+^/NeuN^+^ co-labeled neurons, which were substantially attenuated by EsA administration. Consistently, EsA treatment significantly reduced C-Caspase-3 levels in injured spinal cord tissue, indicating suppression of apoptotic execution. The Bcl-2/Bax family further modulates apoptotic signaling: Bcl-2 antagonizes pro-apoptotic proteins to preserve cell viability, while Bax activates caspases, committing cells to death (38). Accumulating evidence has indicated that the Bcl-2/Bax ratio is a reliable biomarker for assessing neuronal apoptosis following SCI (39). Supporting this notion, EsA reverses the SCI-induced decrease in Bcl-2/Bax ratio by upregulating Bcl-2 and downregulating Bax, thereby preserving neuronal viability. In line with previous reports that EsA has exerted anti-apoptotic effects in Alzheimer's disease (21), our data extended these findings to SCI, demonstrating that EsA confers neuroprotection in our SCI model at least in part through its direct anti-apoptotic activity in spinal cord neurons.

Oxidative stress acts as a key driver of neuronal apoptosis following secondary injury (40). SCI disrupts redox homeostasis, leading to excessive accumulation of superoxide anion (O_2_•^+^) and H_2_O_2_ (41). ROS overproduction induces lipid and protein oxidation, triggering caspase-mediated apoptosis (42). Neurons are particularly vulnerable to ROS attack, since their cell membranes are abundant in polyunsaturated fatty acids (PUFAs) (43). This vulnerability renders them susceptible to lipid peroxidation, resulting in the buildup of cytotoxic byproducts (MDA), which further intensifies neuronal apoptosis (44). Our previous studies demonstrated that reducing H_2_O_2_ and MDA levels effectively attenuates neuronal apoptosis (23). Corroborating prior findings, EsA treatment significantly reduced H_2_O_2_ and MDA levels at key post-injury time points. Organisms have evolved an integrated antioxidant defense system to counteract oxidative stress: SOD dismutates O_2_•^+^ into H_2_O_2_, and GSH-PX reduces H_2_O_2_ to water, thereby preserving cellular integrity (45). Zhang et al. (46) reported that hesperetin attenuated neuronal apoptosis in rats with SCI by upregulating SOD and GSH-PX activities, highlighting the importance of activating antioxidant enzymes for neuroprotection against SCI. Notably, EsA restored SCI-suppressed SOD and GSH-PX activities, with significant improvements at specific post-injury time points. Collectively, these results suggested that EsA alleviated oxidative stress in the injured spinal cord by reducing O_2_•^+^ and H_2_O_2_ production and enhancing antioxidant enzyme activity, which in turn contributed to its anti-apoptotic effects, consistent with the established crosstalk between oxidative stress and apoptosis in SCI (8).

As oxidative stress plays a key part in EsA-mediated neuroprotection, the neuroprotective effects of EsA observed in this study prompted us to investigate its impact on redox-sensitive signaling pathways. Nrf2 orchestrates the cellular antioxidant response, which is crucial for mitigating oxidative damage (47). Specifically, Nrf2 mitigates oxidative damage by promoting the transcription of antioxidant genes (HO-1 and NQO-1), whose protein products either neutralize ROS or maintain cellular redox homeostasis (48). Following SCI, impaired Nrf2 activation compromises endogenous antioxidant defenses, exacerbating neuronal apoptosis, and pharmacological activation of Nrf2 has served as a prospective treatment modality for SCI (49, 50). For example, Phillyrin has been shown to ameliorate SCI by suppressing oxidative damage and apoptosis through modulation of the Nrf2/HO-1 pathway (26). To explore whether the Nrf2 pathway mediates EsA's neuroprotective effects, we first examined the interaction between EsA and the Nrf2/Keap1 complex using molecular docking simulations. *In silico* docking results revealed that EsA formed extensive polar interactions with the Nrf2/Keap1 complex, suggesting a potential mechanism that may contribute to Nrf2 activation and downstream signaling. This was further confirmed by immunofluorescence data showing that EsA treatment enhanced Nrf2 activation in activated microglia, as evidenced by increased Nrf2 expression in CD68^+^/Nrf2^+^ cells within the lesion epicenter. Given that activated microglia produce excessive ROS post-SCI (51), Nrf2 activation in these cells likely mitigates microglia-driven oxidative stress. However, direct evidence linking microglial Nrf2 activation to ROS reduction requires further investigation in subsequent studies. Consistent with these docking and immunofluorescence findings, EsA enhanced Nrf2-mediated HO-1/NQO-1 expression in the injured spinal cord, reinforcing endogenous defense against oxidative stress. This molecular response was strongly correlated with reduced neuronal loss and enhanced locomotor recovery, indicating a potential role of the Nrf2/HO-1 axis in EsA-mediated neuroprotection.

Several natural compounds, such as curculigoside, phillyrin, and the Bu Shen Huo Xue Formula, have been reported to confer protection against SCI by activating the Nrf2/HO-1 pathway (16, 26, 52). These agents have demonstrated the ability to alleviate oxidative stress and neuronal apoptosis in SCI models. Consistent with these findings, EsA also exerted neuroprotective effects by modulating Nrf2-mediated antioxidant activity. Notably, unlike previous studies that primarily focused on the oxidative stress response in neurons, we hypothesize that EsA may exert its neuroprotective effects by activating the Nrf2 pathway specifically in microglia, thereby attenuating microglial-derived oxidative stress. This represents a critical direction for our future investigations. Furthermore, hyperbaric oxygen therapy, a classic clinical intervention for neurological injury, possesses highly consistent core therapeutic mechanisms with EsA. Both strategies can effectively relieve secondary nerve damage and facilitate functional recovery. However, hyperbaric oxygen therapy requires professional equipment and strict treatment conditions, thus limiting its application. As a naturally administered compound, EsA offers greater convenience for widespread use and long-term treatment.

Despite demonstrating the neuroprotective efficacy of EsA, this study still has several limitations. Due to the inherent chemical instability and extremely short half-lives, this study did not include direct quantification of ROS in spinal cord tissue. Future *in vitro* studies will employ an H_2_O_2_-induced microglial-neuronal co-culture system to assess EsA's impact on ROS in both cell types. To confirm that EsA exerts its effects through Nrf2 activation, future *in vitro* studies will include a co-treatment group with ML385 (a selective Nrf2 inhibitor) to verify whether blocking Nrf2 abrogates EsA's beneficial effects. Moreover, several key translational limitations of EsA warrant consideration in future studies. First, the pharmacokinetic characteristics of EsA, including its half-life, tissue distribution, metabolism, and excretion profile, have not been systematically assessed, limiting the rational optimization of administration frequency and therapeutic regimen. Second, the ability of EsA to cross the blood-spinal cord barrier (BSCB) and accumulate at sufficient concentrations in injured spinal cord tissue requires more experimental verification. Third, only a single dose of EsA was used in this study. Further dose-gradient validation is required to determine the optimal therapeutic dosage and effective dose window. Fourth, the long-term safety of EsA administration has not been fully evaluated, and a systematic assessment of its potential hepatic, renal, and neurological side effects is still needed to support its clinical translation. Future studies are warranted to address these issues to facilitate the clinical translation of EsA for SCI therapy.

In summary, our study demonstrated that EsA promoted functional and tissue recovery after SCI by regulating oxidative stress and reducing neuronal apoptosis, a process mediated in part via activation of the Nrf2/HO-1 signaling cascade. Our findings identified EsA as a promising natural product candidate for SCI therapy, and further comprehensive preclinical studies are required to validate its translational potential for SCI treatment in humans.

## Data Availability

The original contributions presented in the study are included in the article/supplementary material, further inquiries can be directed to the corresponding author.
